# Past, Current and Future Techniques for Monitoring Paralytic Shellfish Toxins in Bivalve Molluscs

**DOI:** 10.3390/toxins17030105

**Published:** 2025-02-25

**Authors:** Sarah C. Finch, D. Tim Harwood

**Affiliations:** 1 AgResearch Ltd., Ruakura Research Centre, Private Bag 3123, Hamilton 3240, New Zealand; 2 Cawthron Institute, Private Bag 2, Nelson 7042, New Zealand; tim.harwood@cawthron.org.nz; 3 New Zealand Food Safety Science and Research Centre, Massey University, Private Bag 11 222, Palmerston North 4442, New Zealand

**Keywords:** PST, mouse bioassay, organ-on-a-chip, toxicity equivalence factor, ELISA, receptor binding assay, non-animal methods, 3Rs

## Abstract

Paralytic shellfish poisoning is a threat to human health caused by the consumption of shellfish contaminated with toxins of the saxitoxin class. Human health is protected by the setting of regulatory limits and the analysis of shellfish prior to sale. Both robust toxicity data, generated from experiments fitting into the ethical 3R framework, and appropriate analysis methods are required to ensure the success of this approach. A literature review of in vivo animal bioassays and in vitro and analytical methods showed that in vitro methods are the best option to screen shellfish for non-regulatory purposes. However, since neither the receptor nor antibody binding of paralytic shellfish toxin analogues correlate with toxicity, these assays cannot accurately quantify toxicity in shellfish nor be used to calculate toxicity equivalence factors. Fully replacing animals in testing is rightfully the ultimate goal, but this cannot be at a cost to human health. More modern technology, such as organ-on-a-chip, represent an exciting development, but animal bioassays cannot currently be replaced in the determination of toxicity. Analytical methods that employ toxicity equivalence factors calculated using oral animal toxicity data result in an accurate assessment of the food safety risk posed by paralytic shellfish toxin contamination in bivalve molluscs.

## 1. Introduction

Paralytic shellfish poisoning (PSP) is a worldwide issue caused by the consumption of shellfish contaminated with paralytic shellfish toxins (PSTs). PSTs are toxins that can contaminate filter feeding shellfish that consume the marine dinoflagellates *Alexandrium*, *Gymnodinium* and *Pyrodinium* [1,2,3]. Saxitoxin (STX) is the major toxin of this class, but over 50 structural analogues are known with the ratio of analogues observed being dependent on the microalgae producer and shellfish metabolism [4]. Cases of PSP have been reported throughout the world, including in Norway [5], the UK [6], Canada [7,8], North America [9,10], Chile [11], South Africa [12], Japan and Indonesia [13], Australia [14,15], and New Zealand [15]. The highest number of PSP cases was reported in the Philippines between 1983 and 2002, with 2124 cases and 120 deaths [16]. In Alaska, between 1973 and 1994, there were 54 outbreaks of PSP reported, with 117 cases, 29 emergency treatments and 1 death [9]. PSP outbreaks are characterized by numbness around the lips, muscle weakness and neurological symptoms such as headaches [11]. In severe cases, muscular paralysis will be pronounced, and death may occur due to respiratory paralysis.

To protect human health and facilitate trade, regulatory limits for PSTs must be set, and in order to be effective, they must be made on the basis of robust and accurate toxicity data determined using valid methods. Currently, the European Union (EU) has set a regulatory limit of 800 µg STX.2HCl eq/kg shellfish flesh (regulation (EC) No 853/2004) [17], and the same limit was adopted at the 28th session of the Codex Committee on Fish and Fishery Products (CCFFP) in 2006 [18], resulting in the development of the Standard for Live and Raw Bivalve Molluscs (CODEXSTAN 292-2008, rev 2015) [19]. To adhere to this regulatory limit, shellfish must be analyzed for PSTs using methods with a sufficient sensitivity and with suitable precision and accuracy.

Due to the worldwide enormity of the PSP issue, a wide range of methods for the detection and quantification of PSTs have been developed, ranging from small animal bioassays and in vitro methods to analytical assays. Each of these techniques has strengths and weaknesses making them valuable in some circumstances and not in others. A literature review has been conducted to understand the different methods and allow for the selection of the appropriate method for each specific task. Furthermore, due to increasing pressure to reduce the use of animals in scientific research and the adoption of the 3Rs (reduction, replacement, refinement) by many countries, the role of animals in biotoxin research has been evaluated.

## 2. Methods Used to Determine the PSP Toxicity of Shellfish

### 2.1. Mouse Bioassay (MBA)

The mouse bioassay (MBA) for STX was first developed in the 1930s by Sommer and Meyer [20] and was standardized and issued as the AOAC First Action method in 1959. Subsequently, the method was subjected to a collaborative study that resulted in AOAC Official Method 959.08 for Paralytic Shellfish Poison [21]. The method has been used for many decades and was regarded as the gold standard for detecting PSTs in shellfish in regulatory environments. As such, it has been considered to be an excellent risk management tool to prevent PSP intoxications and facilitate trade. The main advantage of the MBA is that it detects PSTs irrespective of whether their structures have been identified.

The MBA itself is based on the relationship between the amount of pure STX administered by intraperitoneal (i.p) injection to a mouse and “the time elapsed from completion of injection to last gasping of breath”. The relationship is clearly not linear but the slope at death times between 5 and 7 min is close to being linear, and it is this part of the curve that is used. To determine the amount of STX in a solution, mice are injected with various dilutions of an acidified shellfish extract until a death time of 5–7 min is achieved. Through the use of a table portraying the relationship between dose and death time, the amount of STX present (expressed as mouse units per mL) can be determined. One mouse unit is defined as the amount of STX required to kill a mouse in 15 min, and the median mouse units calculated from the MBA can be converted into µg STX/mL using a conversion factor previously determined by calibration experiments.

Despite the PSP MBA being a useful tool to protect health and support industry, there are drawbacks. There are, and will continue to be, ethical concerns around the use of small animals for testing, whether it is for the routine control of shellfish toxins or in other applications, such as research and drug development. There are legislative requirements to pivot away from animal testing in certain parts of the world, as has been well-documented in the EU [22]. For the testing of each shellfish extract, the MBA requires a minimum of three mice. However, to yield a death time in the 5 to 7 min window, multiple dilutions are usually needed, which increases the number of mice used per sample. As evidence, when the PSP MBA was used in the routine monitoring of shellfish in New Zealand, up to 80,000 mice were used per year [23]. The robustness of the method is also of concern. The strain of mouse used has been shown to affect the result [24,25], as has the pH of the extract [25]. In addition, results are confounded for samples that contain high salt concentrations [24,25] and/or elevated levels of metals commonly found in shellfish [26]. The validity of the method has also come under question. The MBA uses an i.p injection, which is not the route of human exposure, and LD_50_s for STX using the two exposure routes differ by orders of magnitude. Furthermore, the magnitude of difference between i.p and oral LD_50_s is not consistent between STX analogues [27]. The MBA also uses death time, which is not a toxicological parameter and is dependent on the dose–death time correlation being the same for each STX analogue. Not surprisingly, this has been shown to be incorrect, with some analogues having different dose–death time relationships to STX [28]. The poor sensitivity of the MBA is another limitation as the detection limit is only approximately half the current PST regulatory limit [29], which is poor when compared to modern instrumental methods. While this detection limit is adequate to determine whether toxin levels in shellfish exceed the regulatory limit, it is unlikely to be sensitive enough to detect the very start of a bloom event or to be able to monitor toxin depuration following bloom collapse.

### 2.2. Analytical Methods

The detection of PSTs is complicated by the variable abundance of over 50 analogues of the parent compound, STX [4]. The initial alternative to the MBA was a method developed in 1975 whereby PSTs were oxidized to fluorescent derivatives and the resultant fluorescent intensity was measured [30]. However, the relationship between analogue toxicity and fluorescence was unknown, meaning that separation of the toxins was required. In 1984, the chromatographic separation and post-column oxidation of PSTs was achieved, an approach colloquially known as the Oshima method [31]. Further improvement was achieved using a post-column derivatization liquid chromatography method [32]. A comparison of results derived using this analytical method with those derived using the MBA showed a reasonable correlation, although the MBA yielded lower values [32]. An alternative separation and analysis system using capillary electrophoresis coupled to mass spectrometry was studied, but this method had limitations in separation efficiency and was restricted to low sample volumes [33]. Interest then turned back to chromatographic approaches, this time utilizing the pre-column oxidization of shellfish extracts prior to fluorescence detection. Various iterations of this method were published until Lawrence et al. [34] derived a method that showed good repeatability and interlaboratory reproducibility as well as a good correlation with the MBA. This method, colloquially referred to as the Lawrence method, became an official AOAC method (2005.06) [35]. In addition to the Lawrence method, the post-column oxidization process [32] was also subjected to comprehensive inter-lab validation [36]. The results showed good reproducibility and, as such, in 2011, this post-column detection method was also accepted by the AOAC for the testing of PSTs in a number of bivalve species (official method 2011.02) [37]. However, the throughput, detection limit and complexity of the above-mentioned analytical methods were problematical (e.g., for the Lawrence method, complexity is encountered through the high number of sequential steps required to analyze positive samples, and there is difficulty in interpreting the data when multiple PST analogues are present). As an alternative, a method using hydrophilic interaction chromatography (HILIC) linked with tandem mass spectrometry (HILIC-MS/MS) was trialed, but high detection limits and substantial sample matrix issues prevented its use as a high-throughput approach [38]. However, Boundy et al. [39] overcame these issues using a novel sample preparation approach, resulting in a quick, selective and sensitive quantitation method for the analysis of PSTs from many bivalve species of shellfish [39].

#### Toxicity Equivalence Factors (TEFs)

Analytical methods allow for the chromatographic separation and accurate quantification of PSTs in different shellfish types. However, the concentration of PSTs in a sample cannot be related to the regulatory limit without an adjustment to account for the differences in analogue toxicity, which in the case of PST analogues can be considerable. This adjustment can be made by applying toxicity equivalence factors (TEFs) [40]. TEFs are defined as “the toxicity ratio of a compound from a chemical group that shares the same mode of action of a reference compound of the same group” [41]. The toxicity of each PST analogue is expressed as a fraction of the toxicity of STX, and the application of these experimentally derived TEFs to the concentrations determined analytically allows the toxin content of samples to be expressed as STX equivalents (“STX eq”). Once converted to STX equivalents, the concentrations of the analogues can be totaled, yielding a figure that can be used to assess sample toxicity and therefore the risk they pose to human health. Although the performance characteristics of analytical methods are important and can be ascertained, to generate a robust estimation of sample toxicity, it is also essential that the TEFs applied are accurate.

**Figure 1:**
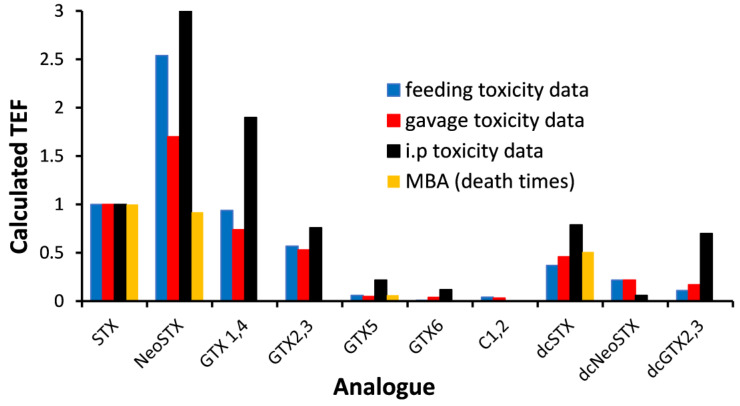
Toxicity equivalence factors (TEFs) determined for STX analogues; neosaxitoxin (NeoSTX), gonyautoxins 1&4 (GTX1&4), gonyautoxins 2&3 (GTX2&3), gonyautoxin 5 (GTX5), gonyautoxin 6 (GTX6), N-sulfocarbamoyl gonyautoxins 2&3 (C1&2), decarbamoyl STX (dcSTX), decarbamoyl neoSTX (dcNeoSTX), decarbamoyl gonyautoxins 2&3 (dcGTX2&3) using data determined by the voluntary feeding [28,41], gavage [28,41], or intraperitoneal injection [28,43] of pure toxins to mice. Data generated by the MBA, where available, are also presented [32].

PST analogue toxicity data can be obtained using a number of different methods. As discussed previously, the MBA is not a true measure of toxicity since death time is not a toxicological parameter. The determination of a median lethal dose (LD_50_) is more appropriate, but this can be determined using different routes of administration, which can have a large effect on the TEF value assigned (Figure 1). An i.p injection represents an artificial situation, unlike that within the gastrointestinal tract, with the injected compounds being rapidly and effectively absorbed from the peritoneal cavity. Furthermore, i.p injection bypasses first-pass metabolism, which may impact compound bioavailability and therefore toxicity. Administration by gavage represents an improvement since the test compound is applied directly in the stomach, but gavage is a difficult technique to perform and can often result in rapid death due to the aspiration of toxins into the lungs. Additionally, it has been shown, using a radiolabeled protein, that 38% of mice dosed by gavage did not receive their appropriate liquid dose, which would result in an underestimation of toxicity of the administered compound [42]. Voluntary feeding is the most appropriate dosing method as it ensures that the dose is swallowed naturally and reduces stress on the animal. The approach of mixing the test compound with a very small amount of cream cheese (150 mg) has previously been used to successfully administer a range of compounds, including those of marine origin. Toxicity is unaffected by the cream cheese matrix, and mice eat the laced cream cheese quickly, within 30 s [27].

A comparison of TEFs derived using mice obtained by the different dosing methods shows that those determined by i.p injection are higher for a range of STX analogues (Figure 1). However, the magnitude of difference between the i.p TEFs and those determined by other methods is not consistent, meaning that oral toxicity cannot be predicted on the basis of i.p toxicity. TEFs generated by gavage administration also showed some differences from those obtained by feeding. Of most concern were the TEF values for neosaxitoxin (NeoSTX), which were 1.70 by gavage and 2.54 by feeding. For a sample containing NeoSTX, the use of the TEF generated by gavage would result in a considerable underestimation of toxicity. In addition to the practicalities associated with gavage dosing, it has been well-documented that this method can overestimate the toxicity of shellfish toxins. This is thought to be due to the semi-solid nature of the mouse stomach contents, which allow a liquid gavage dose to flow around the mass to be rapidly absorbed by the duodenum [28,41]. In contrast, the ingestion of the dose with a solid matrix (cream cheese) allows for effective mixing with the stomach contents. TEF values using LD_50_ data generated by feeding mice were compared to those generated using the death times of mice injected with toxins in an MBA. This showed some significant differences—for example, TEFs for NeoSTX were 2.54 and 0.92 by feeding mice and by using the MBA, respectively (Figure 1). This is not unexpected given the inherent issues associated with the MBA.

In 2009, the EFSA proposed TEFs for PST analogues based on acute toxicity determined by the i.p injection of mice. However, the CONTAM (The Panel on Contaminants in the Food Chain) Panel noted that further toxicological data were needed to establish robust TEFs and that these values should be set on the basis of acute oral toxicity data [44]. In 2016, the FAO/WHO panel updated TEF values for any analogues where additional data were available, with preference given to TEFs derived orally [41].

Despite more toxicity information now being available for STX analogues allowing updated TEFs to be calculated, those proposed in 2016 are still used. To change TEF values, a scientific panel must be convened to consider all new information and make recommendations.

### 2.3. Non-Animal Methods

Although the determination of TEFs requires only a fraction of the mice used in the MBA, the use of animals in research and regulatory testing has come under increased pressure. The concept of the 3Rs (reduction, refinement and replacement) in animal research was first proposed by Russell and Burch [45] in 1959. Reduction is defined as “a means of lowering the number of animals used to obtain information of a given amount and precision, without increasing the welfare costs to individual animals”. Refinement is defined as “any development leading to a decrease in the incidence or severity of procedures applied to those animals that have to be used”, and replacement is defined as “the use of any scientific method employing non-sentient material, which could replace procedures that use conscious living vertebrates” [46]. In 2010, the EU legislation covering the use of animals was updated to improve the welfare of animals and to incorporate the 3Rs, with this legislation coming into effect in 2013 (EU legislation directive 2010/63/EU). An enormous amount of time and money has been invested into the development of novel approach methods (NAMs) as alternatives to the use of animals. In 2009, the European Commission made a call for proposals as part of the 7th Framework Programme (FP7) Research Initiative, and EUR 50 million was invested in progressing non-animal test methods [22]. In 2011, the Safety Evaluation Ultimately Replacing Animal Testing (SEURAT) initiative implemented projects involving 70 European universities, public research institutes and enterprises, and in 2016, an additional EUR 30 million was invested by the Integrated European Flagship Programme Driving Mechanism-Based Toxicity Testing and Risk Assessment for the 21st century (EU-ToxRisk) [22]. Internationally, there is a commitment to the 3R principles, and in 2000, the Interagency Coordinating Committee on the Validation of Alternative Methods (ICCVAM) was established, which works alongside the National Toxicology Program’s Interagency Center for the Evaluation of Alternative Toxicological Methods (NICEATM). These organizations also interact with the Organisation for Economic Co-operation and Development (OECD), forming an alliance involving the United States, Europe, Japan, Korea and Canada (International Cooperation on Alternative Test Methods (ICATM)) to promote the development, validation and regulatory acceptance of non-animal models [47]. In 2021, the European Parliament adopted a resolution to “accelerate a transition to innovation without the use of animals in research, regulatory testing and education” [48], suggesting that animal testing could be completely eliminated.

#### 2.3.1. Cell-Based Assays

**Figure 2:**
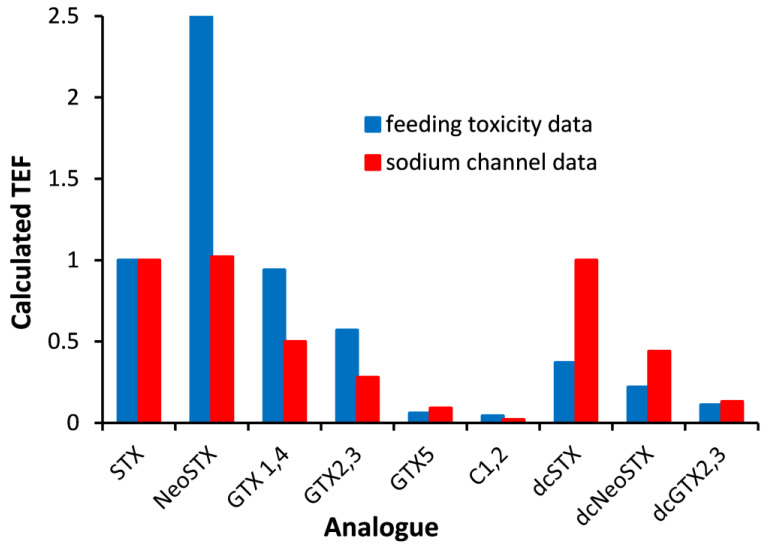
Toxicity equivalence factors (TEFs) determined for STX analogues; neosaxitoxin (NeoSTX), gonyautoxins 1&4 (GTX1&4), gonyautoxins 2&3 (GTX2&3), gonyautoxin 5 (GTX5), N-sulfocarbamoyl gonyautoxins 2&3 (C1&2), decarbamoyl STX (dcSTX), decarbamoyl neoSTX (dcNeoSTX), decarbamoyl gonyautoxins 2&3 (dcGTX2&3) using data determined by the Neuro-2A assay [63] in comparison to those determined by feeding mice [28,41]).

PSP is induced by the blockage of sodium channels [49], and in 1988, Kogure, et al. [50] developed a tissue culture assay using the mouse neuroblastoma cell line (Neuro-2A). The presence of a sodium channel activator (veratridine) allows for an influx of sodium ions into cells, and with the addition of ouabain, a blocker of sodium ion efflux, Neuro-2A cells swell and die. PSTs, as sodium channel blockers, reverse this effect by preventing cell death in a dose-dependent manner. Improvements to the original assay were made in 1993 by using crystal violet [51] or a tetrazolium salt (MTT) [52] to measure cell growth and viability. An evaluation of this assay showed it to have good sensitivity, and the comparison of PST concentrations in shellfish determined by the MBA and the Neuro-2A assay showed a good correlation (r = 0.90), although the Neuro-2A assay gave higher concentrations [53]. In 1996, following similar methods, a bioassay kit—the Maritime In Vitro Shellfish Test (MIST™)—was developed by Jellett Biotek Ltd., Dartmouth, NS, Canada [54]. This kit was available in three formats: quantitative, semi-quantitative and qualitative (yes/no). However, when tested in an AOAC International collaborative study in 1999, the procedure did not yield satisfactory results, leading to its discontinuation [43]. Limitations of the Neuro-2A assay included matrix effects and a relatively long analysis time (24–48 h). The analysis time was reduced to 8 h using a modified format using brevetoxin to enhance sodium influx into the cell [55]. Following the same concept, maitotoxin, a calcium agonist, was used to decrease cell death, which further reduced the analysis time [56]. The comparative analysis of 34 shellfish samples using the latter assay and the MBA showed PST results with a good correlation (Spearman rank-order correlation coefficient (r) of 0.95). Further assay development was made using voltage-sensitive dyes which, rather than measuring MTT induced cell death, measured changes in the nerve cell membrane potential induced by PSTs [57,58]. A more comprehensive evaluation of the Neuro-2A assay was conducted by evaluating PSTs in 367 shellfish samples collected from Japan [59]. In this study, a different tetrazolium salt was used (WST-8), which, unlike MTT, is water-soluble, making it easier to use. The results from the cell bioassay and LC-analysis method showed a good correlation (r = 0.90), but the cell assay gave a negative bias, as indicated by the slope of the regression line (0.71). In a further variation of the Neuro-2A assay, a cell-based biosensor was developed. This method allowed for the real-time, non-invasive monitoring of the growth status of in vitro cultured cells, showing good sensitivity and specificity for STX [60]. In 2015, an additional impedance biosensor was developed using cardiomyocytes, which showed STX to inhibit beating in a concentration-dependent manner [61]. However, this method was less sensitive to TTX, which has been shown to have the same mechanism of action and the same toxicity to mice as STX [62]. With the availability of PST standards, the relative sensitivity of the analogues was assessed using the Neuro-2A assay. TEFs could be determined by calculating the ratio between the EC_50_ (the concentration that gives a half-maximal response) of STX and the EC_50_ determined for each analogue [63]. TEFs derived by oral toxicity data using mice are considered to be the most valid, and comparisons with those generated by the Neuro-2A assay showed significant inconsistencies (Figure 2).

The most obvious difference in the TEFs derived by the Neuro-2A assay and those derived using oral mouse toxicity data were those for NeoSTX which were 1.24 and 2.54, respectively. TEFs generated by mouse toxicity data were also higher for GTX 2&3 and dcNeoSTX. This means that the Neuro-2A analysis of samples containing these analogues would underestimate toxicity.

#### 2.3.2. Receptor Based Assays

Since receptor binding assays involve measuring the effect of toxins on the mechanism of action, it was assumed that the relative binding affinities of the STX analogues would be correlated to toxicity, thus conferring greater accuracy compared to other approaches. Using sodium channels derived from rat brain membranes, a solid-phase radioreceptor assay (RBA) was developed [64]. In this assay, PSTs could be quantified based on their ability to competitively displace ^3^H-STX from the sodium channel receptor site. The spiking of STX into mussel extract showed a 95–120% recovery using this assay. Furthermore, the analysis of PST mixtures by HPLC (post-column derivatization, Oshima method), MBA and RBA showed that the consistency between the HPLC and RBA results was closer than that between HPLC and the MBA [64]. Efficiency was improved by adaption to a microtiter filter plate-based version of the receptor assay. The analysis of algal and shellfish extracts by HPLC and RBA showed a good consistency, with correlation coefficient (r) values of 0.88 and 0.97, respectively [65]. However, when the relative binding affinities of PSTs using the RBA were compared with TEFs generated using in vivo data, some large differences were observed. For example, TEFs of 0.5 and 0.1 were determined for dcSTX using in vivo [32] and RBA data [66], respectively. In addition, TEFs of 0.06 and 0.03 were determined for GTX5 using in vivo and in vitro data, respectively [66,67]. Despite this result, interest in the RBA remained high, and a method for PSTs was validated through an interlaboratory comparison that showed it to have high throughput and good reproducibility—desirable traits for routine monitoring purposes [68]. However, the analysis of naturally contaminated samples by RBA and MBA showed that PST concentrations were higher when determined by RBA [68]. A further collaborative study involving nine laboratories from six countries yielded results of sufficient quality for this method to be accepted as an AOAC official method for the detection of PSTs in shellfish (mussels, clams and scallops) (AOAC official method 2011.27) [69]. Although the method yielded higher STX eq concentrations than the MBA, it was closely correlated to those generated by the pre-column oxidation HPLC method (slope of 1.20 and r^2^ of 0.92). The RBA method was also accepted in the United States of America (US) for certain applications but was not used in the EU due to restrictions around the use of radioactive material [41]. Although the AOAC RBA method was approved in the US for the analysis of mussels, clams and scallops, Turner, et al. [70] compared the performance of the RBA method with the pre-column oxidation HPLC method (AOAC official method 2005.06) using a wider range of shellfish species sourced from Great Britain. This study showed that the RBA tended to overestimate PST concentrations in naturally occurring shellfish, with 18% of samples analyzed by RBA showing results more than double the total PST concentrations determined analytically, with the majority of these samples being oysters (75%) [70].

An alternative RBA was developed using ^3^H-STX binding to saxiphilin, a high-affinity STX-binding protein found in circulatory fluids of certain organisms [71]. Analyses of shellfish for PSTs using this method were compared to those determined using the post-column derivatization HPLC method. Although this showed the two methods to give reasonably consistent results, the saxiphilin assay gave both false positives and negatives. The unavailability of saxiphilin also meant that this assay was not developed further [71].

**Figure 3:**
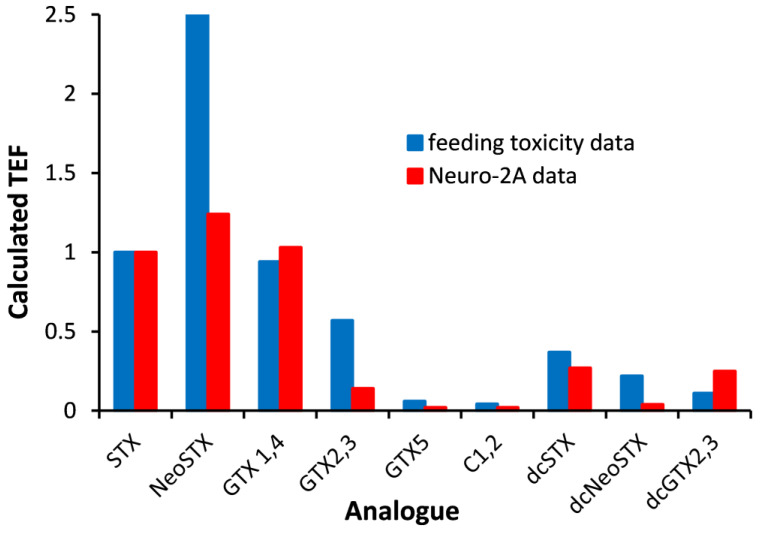
Toxicity equivalence factors (TEFs) determined for STX analogues; neosaxitoxin (NeoSTX), gonyautoxins 1&4 (GTX1&4), gonyautoxins 2&3 (GTX2&3), gonyautoxin 5 (GTX5), N-sulfocarbamoyl gonyautoxins 2&3 (C1&C2), decarbamoyl STX (dcSTX), decarbamoyl neoSTX (dcNeoSTX) and decarbamoyl gonyautoxins 2&3 (dcGTX2&3) using data determined using voltage-gated sodium channels in cultured neurons [73] and using toxicity determined by feeding mice [28,41].

Since binding to a cell does not reflect how PSTs impair sodium channel function, work was conducted to develop an electrophysiological assay using cultured neurons. Single cells expressing STX-sensitive sodium channels could be patch-clamped, and extracts could be applied and electrophysiological recordings collected. Using this method, it was shown that PST concentrations were well-correlated to those determined by the standard RBA, albeit with enhanced sensitivity [72]. However, Perez, et al. [73] determined TEFs using this method, which showed them to be inconsistent with their toxicity (Figure 3). Compared to the TEFs determined using in vivo toxicity data, those of particular significance were the much lower TEFs generated for NeoSTX and GTX1&4 by the RBA along with the considerably higher TEF generated for dcSTX.

Mammals have 10 sodium channel subunits denoted as Na_v_1.1-1.10. Na_v_1.2 and Na_v_1.6 are the major isoforms expressed by cerebella neurons such as those used in the above study, but Na_v_1.1 and Na_v_1.3 are also neuronal-type subunits, Na_v_1.4 is present in skeletal muscle, Na_v_1.5 is a cardiac-specific subunit and Na_v_1.7 is located in the peripheral nervous system. Using human cell lines and automated patch clamp electrophysiological recordings, Alonso, et al. [74] determined the activity of PSTs on each of the individual sodium channel subunits (Table 1). TEFs determined for the Na_v_1.1, 1.5 and 1.7 subunits showed no consistency with those generated from in vivo feeding toxicity data. TEF values derived on the individual sodium channels were expressed as a percentage of those generated from in vivo data, showing that each of the subunits is better for some of the PST analogues and worse for others. Interestingly, the Na_v_1.2, 1.3 and 1.4 subunits gave an accurate TEF for NeoSTX. However, Na_v_1.2 gave a low TEF for all of the other PST analogues tested, Na_v_1.3 gave a low TEF for dcSTX (22%) but high TEFs for GTX1&4 (170%) and dcGTX2&3 (200%), and Na_v_1.4 gave a very high TEF for GTX5 (300%) and a low TEF for other analogues such as dcNeoSTX (0.5%). Na_v_1.6 not only gave a low TEF for NeoSTX (47%); it also gave high TEFs for other PST analogues such as dcSTX (260%), GTX5 (183%) and GTX1&4 (149%). A comparison between TEFs generated from i.p toxicity data and those derived from sodium channel activity also showed no consistency. As observed in the comparison between TEFs from in vivo feeding, there was no consistency between PST analogues on the same subunits, with some being very high and some low. For example, compared to the i.p in vivo TEF, those determined on the sodium subunit 1.2 were 167 and 6% for dcNeoSTX and GTX5, respectively.

In a further study, the toxicity of dcGTX1&4 determined by feeding mice was compared to activity on the Na_v_1.4, 1.5 and 1.7 cell lines using a voltage-gated sodium channel activity fluorescent imagine plate reader (FLIPR) assay [75]. dcGTX1&4 showed activity on only the Na_v_1.4 subunit, although the generated TEF (<0.01) was not comparable to that determined by feeding mice (0.09) [75].

#### 2.3.3. Immunological Based Assays

Immunological assays can be developed using antibodies that recognize certain structural features of the target molecule. The success of this approach is dependent on the properties of the antibodies and their ability to recognize multiple structures. This is particularly complicated for the PST analogues due to the large number of structural variants. For accurate toxicity information to be generated using these techniques, the cross-reactivities of the different analogues to the antibodies must reflect the in vivo toxicity of each analogue.

##### Enzyme-Linked Immunosorbent Assays and Lateral Flow Assays

An ELISA developed in 1985 by Chu and Fan [76] showed high affinity for STX but cross-reactivities of 56 and 16% for dcSTX and NeoSTX, respectively. An enzyme-linked immunosorbent assay (ELISA), developed in 1984 [77], was commercially released as a kit (Ridascreen, R-biopharm, Darmstadt, Germany) but was shown to underestimate the toxicity of shellfish samples in Japan [78]. This issue was caused by the kit having low cross-reactivities to gonyautoxins (0.1–0.55%), which are particularly prevalent in Japanese shellfish. In Japan, it was therefore thought that “the MBA cannot be replaced with the ELISA kit for the purpose of screening inshore shellfish samples” [78]. Due to the simplicity and quick analysis time achievable with ELISAs, further development was undertaken by many groups around the world, but progress continued to be hampered by the inability of this technique to adequately assess the entire toxin class. In an attempt to overcome these limitations, separate antibodies were raised against STX and NeoSTX. Although attempts to combine both antibodies in the same plate format were unsuccessful, samples could be analyzed using each antibody separately and then summing the results. Analysis of 1540 PST contaminated samples using this dual antibody approach showed 13.6% to be over the regulatory limit compared to 11.3% detected by the MBA [79]. An alternative approach was to develop an ELISA based on an antibody raised against GTX2&3 [80]. This ELISA had higher cross-reactivities to GTX2&3, dcGTX2&3, C1&C2 and dcSTX compared to those for GTX1&4 and NeoSTX. The analysis of Japanese shellfish samples using this ELISA showed it to overestimate PSTs. Each ELISA was developed using different antibodies, meaning that their cross-reactivities to PST analogues were different. Since PST profiles can vary greatly depending on the microalgal species involved, to ensure there is no under- or overestimation of sample toxicity, it is important to understand which detection method is best suited to the analogues present in the samples. For test kits to accurately determine sample toxicity, the cross-reactivies must mirror in vivo toxicity.

In a variation to ELISAs, lateral flow devices can be used to yield a yes/no result as to whether samples exceed the PST regulatory limit (800 µg/kg STX (eqs)). However, since these assays are also based on the recognition of a chemical structure by antibodies, they suffer with the same cross-reactivity issues as those encountered by ELISAs. Jellet Biotech used a mixture of antibodies to allow for the detection of both STX and NeoSTX derivatives, although the cross-reactivity of the assay to NeoSTX was still low. The resulting assay, MistAlert™, was tested on 2100 shellfish samples from different areas of the world. Not surprisingly, the performance of the assay depended on the toxin profile of the samples, with 5% of samples containing concentrations of PSTs < 40 µg/g testing positive and 5% of samples contaminated with 40–80 µg/g PSTs testing negative [81]. Nevertheless, in 2003, this assay was approved for use as a shellfish screening tool in the US [82]. Further testing of shellfish samples collected in 2003–2007 showed that assay performance was again dependent on the toxin profiles [83]. A further PST lateral flow immunoassay was developed by Neogen. The analysis of a range of shellfish samples using this device showed a good correlation to those generated by the MBA method and by the pre-column oxidation (Lawrence) HPLC method [84]. Neogen discontinued their rapid kits for marine toxin analysis in 2024, including a kit for PSTs (Reveal 2.0 for PSP).

In 2016, Harrison, et al. [85] assessed six commercially available PST rapid test kits, including lateral flow assays (LFAs) and ELISAs, by comparing the results of each to those generated using instrumental analysis. The lateral flow device, Neogen, gave few false negatives (2%) but more false positives (25%). Two of the five ELISAs tested, Europroxima and Bioscientific-MaxSignal assay (Bioo), were found to have an inappropriate linear range and underestimated toxicity by a factor of 0.17 and 0.65 compared to the HPLC analysis using the Lawrence method. In contrast, two of the ELISAs, Beacon and Abraxis, overestimated toxicity by factors of 1.90 and 1.32, respectively. The remaining ELISA, R-biopharm (Ridascreen), produced results that were most comparable to the HPLC analysis (95%). The difference in performance of the assays is not surprising since each is based on antibodies raised independently and as such poses different cross-reactivities to the various PST analogues that are present in naturally incurred samples. Cross-reactivities and a comparison to TEFs derived from in vivo toxicity data for the different test kits are presented in Table 2. Data for MistAlert™ (later renamed Scotia) are also included in the table.

Table 2 clearly shows the vastly different PST recognition abilities of the rapid test kits and demonstrates how the cross-reactivities are inconsistent with in vivo toxicities. The recognition of NeoSTX and GTX1&4 was low for each of the test methods, with the Neogen kit being closest to the TEF derived using in vivo toxicity data (51 and 6.4%, respectively). In comparison, the cross-reactivity of GTX5 was high for all kits assessed (383–1017% of the in vivo TEF). The impact of these assay characteristics would mean that if shellfish samples contain NeoSTX or GTX1&4 then toxicity would be underestimated, whereas if GTX5 was present then toxicity would be overestimated.

To improve the accuracy of the immunoassays, an incubation step with L-cysteine was proposed. This step converts GTX2&3 and GTX1&4, which have low cross-reactivities that do not reflect their in vivo toxicity, to STX and NeoSTX, which have higher cross-reactivities [86]. The testing of mussels and oysters from Tasmania showed that the Abraxis and Europroxima test kits performed poorly, with an underestimation in toxicity [87]. The two lateral flow immunoassays performed better, with Scotia giving no false negatives and 27% false positives and the Neogen kit giving 5% false negatives and 13% false positives. Furthermore, the addition of the L-cysteine step could be used to eliminate the false negatives [87]. A larger interlaboratory study of the Neogen assay involving 16 laboratories in eight countries was conducted [88]. Using the conversion step with L-cysteine, the Neogen kit was shown to have an acceptable performance for oyster samples, but the case was less convincing for mussels [88]. In an attempt to provide the shellfish industry with a quick screen for different classes of toxin, a multiplex lateral flow assay was developed [89]. This assay used a reader to provide a quantitative assessment of samples rather than just the qualitative (yes/no) result traditionally associated with lateral flow assays. In addition to the detection of okadaic and domoic acids, the kit detected STX with a working range applicable to the PST regulatory limit. Spiking mussel extracts with STX showed a 131% recovery, but no other PST analogues were tested [89].

##### Biosensors

Biosensors are devices with recognition molecules that are immobilized and in contact with a signal generation element (i.e., a transducer). The transducer generates a signal that is proportional to the concentration of the analyte. A large variety of biosensors are available and many have been developed and trialed for the detection of PSTs [90]. Biosensors are classified according to their transducer detection principles and include optical devices, electrochemical sensors, electrochemiluminescence, field-effect transducers and acoustic devices. An optical biosensor base on surface plasmon resonance (SPR) was developed into a kit format that underwent an inter-lab study involving six participants, showing a good repeatability between laboratories [91]. However, while the accuracy of the kit was acceptable for STX, its performance was less reliable when other PST analogues were present. No consistency was observed between results generated using this biosensor, the MBA and HPLC using the Lawrence method. This would have been caused by the cross-reactivities of analogues to the biosensor antibodies. In this case GTX5, C1&C2 and GTX1&4 had cross-reactivities relative to STX—of 1300, 217 and <1.1%, respectively [91]. In comparison, an SPR biosensor using an antibody raised against GTX2&3 was tested against PSTs and naturally contaminated shellfish samples [92]. This showed that the cross reactivities of dcSTX, GTX2&3, GTX5 and C1&C2 were slightly higher than that of STX and that NeoSTX, GTX1&4 and dcNeoSTX could not be detected. The analysis of natural samples alongside the MBA and HPLC (Lawrence method) showed the SPR assay to overestimate toxicity by a factor of at least five [92]. Another form of biosensor, this time utilizing magnetic electrochemical detection, was developed to detect STX. This biosensor was ultra-sensitive to STX but showed no response to GTX1&2 [93]. In 2014, GE Healthcare (Uppsala, Sweden) developed two multiplex biosensors using the Biacore platform (Biacore Q and prototype multiplex, both with SPR detection) to simultaneously detect shellfish toxins of different classes, STX, okadaic acid, domoic acid and palytoxin [94]. Although testing showed that the Biacore Q method was not suitable, the prototype detected all toxin types with adequate sensitivity. However, TEFs generated on the basis of antibody specificity were 203, 500, 983, 11, 37, 35 and 22% of those generated using in vivo feeding data for NeoSTX, dcSTX, dcNeoSTX, GTX5, GTX2&3, dcGTX2&3, C1&C2 and GTX1&4, respectively. These figures show that the binding of analogues to the biosensor antibody is not correlated to toxicity. Furthermore, the methodology was of high cost, and it appears that it was not further developed.

Aptamers are also a form of biosensor, but rather than using antibodies raised against specific targets, they use single-stranded oligonucleotide sequences (DNA or RNA) obtained from Systematic Evolution of Ligands by Exponential enrichment (SELEX). The SELEX process results in aptamers with high affinity and specificity towards the molecules used in their generation. The first aptamer developed for the detection of marine toxins was that of Handy, et al. [95], which, using SPR detection, showed a concentration-dependent selective binding of STX. Many aptamers have been developed for PSTs utilizing multiple different detection systems [96]. For example, an optical aptasensor for the specific detection of GTX1&4 showed no cross-reactivity to GTX2&3, STX or NeoSTX [97]. An electrochemical aptasensor for the detection of STX in seawater also showed no cross-reactivity to GTX1&4 or NeoSTX [98]. Similarly, a colorimetric aptosensor showed no cross-reactivity to GTX1&4 or NeoSTX but gave a good recovery of STX spiked into seawater or scallop extracts [99]. Compared to traditional methods of raising antibodies, aptamers do not require animals, are of low cost and offer a flexibility of format. However, since aptamers are like a monoclonal antibody, they bind with high specificity to the target compound used in their generation but have cross-reactivities to other analogues that are not correlated to their toxicity. This makes aptamers, in their current form, unsuitable for the detection of an entire toxin class.

#### 2.3.4. Other Detection Systems

Zebrafish (*Danio rerio*) larvae have been used extensively in drug screening as they have functional livers, kidneys, and blood–brain barriers, as well as drug-metabolizing enzymes and metabolic rates that are comparable to humans [100]. Unlike cell assays, this model can therefore provide information on the absorption, distribution, metabolism and excretion (ADME) of compounds. A bioassay to detect PST and amnesic shellfish toxins has been developed by measuring motor behavior impairments induced on larvae [101]. STX induced a concentration-dependent decrease in locomotor activity as well as changes in survival, periocular edema, body balance and touch response. Although TEFs for other PST analogues were not reported, the order of potency was given as NeoSTX > STX = dcSTX > GTX1&4 > GTX2&3 > C1&2 [101]. In contrast, toxicity determined by feeding mice shows dcSTX to be less potent than both GTX1&4 and GTX2&3 [28]. An assay for the detection of PSTs in shellfish flesh has also been developed using desert locusts (*Schistocerca gregaria* L.) [102]. In this assay, locusts were injected with shellfish extracts, and the resulting paralysis was calibrated against responses generated by pure STX. The calculation of TEFs using data derived by this method for NeoSTX, dcSTX and GTX2&3 gave values of 0.56, 0.27 and 1.63, respectively [102]. In comparison, TEFs generated using toxicity data from mice fed the same compounds were 2.54, 0.37 and 0.57, respectively. This demonstrates no consistency between the relative potencies of PST analogues determined in the locust and mammalian systems. Bernardi Bif, et al. [103] showed a dose–response relationship between the concentration of STX and the mortality of *Mysidopsis juniae*. Furthermore, STX induced abnormalities in the development of appendices of *Lytechinus variegatus* and *Arbacia lixula* sea urchin larvae [103].

### 2.4. Assays Used for Regulatory Control of PSTs

Historically the MBA has been used worldwide to screen shellfish for PSTs. However, as described above, there are now a myriad of testing tools available, but unfortunately, there is no consistency in their uptake throughout the world. Although not a comprehensive list, a snapshot of monitoring approaches used in different regions of the world has been collated.

In New Zealand, the use of the MBA (AOAC 959.08) ceased in 2010 in favor of analytical methods. Initially the pre-column oxidation with fluorescence detection method (AOAC 2005.06; the Lawrence analytical method [34]) was used, which was found to be fast and sensitive for screening shellfish extracts for the presence of PSTs. However, when needing to confirm the contribution of each analogue to the total toxicity of the sample the method was cumbersome and expensive making it untenable in the New Zealand setting. An alternative quantitative LC-MS/MS analytical method was developed in collaboration with scientists from Cefas in the United Kingdom (UK). This method, ‘PSP by HILIC-MS/MS’ [104], is colloquially known as the Boundy method and was internationally validated using many different shellfish species and toxin profiles from around the world. The Boundy method was approved for the regulatory control of PSTs in New Zealand in 2015 and has been used successfully since this time. No commercially available rapid test kits can be used for the regulatory control of PSTs in New Zealand. However, businesses are free to use them within their own operations to augment their PST monitoring obligations, and there remains interest in their use to support real-time shellfish harvesting decisions. Australia has followed a similar path to New Zealand, with the MBA being phased out in preference of analytical instrumental methods. Initially, this was the Lawrence method, which was adopted in 2012, and then the ‘PSP by HILIC-MS/MS’ method was also included as an approved method in 2020. In contrast to New Zealand, there is also provision in Australia for the different state competent authorities to use commercially available rapid testing kits for PST monitoring under some circumstances, contingent on them being validated to an acceptable standard.

In Europe, which at the time included the United Kingdom, legislation was changed in 2006 to allow the Lawrence analytical method (AOAC 2005.06) to be considered an alternative method to the MBA for the detection of PSTs in bivalve molluscs [105]. Since this time, the method has been refined, validated and implemented into a number of official control monitoring programmes within European member states, and in 2019, it became the official EU reference method for PST determination in shellfish (BS EN 14526) [106]. EU legislation was amended in 2021 to remove the use of live animals for PSP monitoring (EU 2021/1709), with the wording allowing ‘any other internationally recognised method not entailing the use of live animals’ to be used, although, to our knowledge, this has not been applied to date. The United Kingdom withdrew from the EU in 2020 and is no longer bound by this legislation for national controls, so it has the ability to independently choose what methodology is used. However, the United Kingdom needs to continue to demonstrate equivalence of controls to maintain exports to the EU, so, to date, no changes have been made and, along with the majority of Europe, it continues to use the Lawrence method (AOAC 2005.06) for the regulatory monitoring of PSTs. Rapid test methods are not able to be used in the EU for official control purposes, but they are actively recommended in the United Kingdom for end-product testing.

In Japan, the MBA remains as the current official testing method for PSTs. A lateral flow immunoassay method that was developed in Japan is able to be used to re-open shellfish harvesting in a particular growing area located in the Osaka prefecture [107]. In addition, a post-column oxidation fluorometric HPLC method is able to be used for PST monitoring in some areas [108]. The lack of PST reference materials in Japan has hindered the transfer of testing from the MBA to analytical methods. Current research is determining detailed PST profiles in Japanese shellfish, with the aim of allowing the introduction of LC-MS/MS for regulatory monitoring in a few years.

In the United States, the situation is more complex, with the MBA (AOAC 959.08), receptor binding assay (AOAC 2011.27), and post-column oxidation with fluorescence detection method (AOAC 2011.02) all being approved methods for PST monitoring. Details of approved methods are outlined within the National Shellfish Sanitation Program (NSSP), which is the federal and state cooperative program recognized by the U.S. Food and Drug Administration (FDA) and Interstate Shellfish Sanitation Conference (ISSC) for the sanitary control of shellfish produced and sold for human consumption [109]. Of the approved methods, most laboratories still use the MBA, although FDA research laboratories remain aware of new methods such as the LC-MS/MS. Commercially available rapid test kits are able to be used for some specific applications. The Abraxis Shipboard ELISA can be used for PST screening and is employed as a pre-harvest screening tool using a lot-testing monitoring strategy, which examines a batch of products to ensure they meet quality standards and specifications. The AquaBC lateral flow assay is mainly used as a screening tool to maintain an area in the open status, determine when to perform an MBA, and to make precautionary closures.

In South America (e.g., Chile and Argentina), the MBA is used as the primary monitoring tool for the regulatory control of PSTs. Testing using the MBA in Chile, a large producer of bivalve shellfish, is regarded as highly efficient with shellfish being able to be certificated for consumption within 4 h upon receipt at the laboratory. The Lawrence analytical method is also approved in Chile for testing exported shellfish. To the best of our knowledge, commercially available rapid test kits are not able to be used for the regulatory control of PSTs in South America.

## 3. Potential Future Testing Options

The use of animals for testing the toxicity of chemicals, including toxins, raises ethical concerns, and considerable effort has been dedicated to the development of new approach methodologies (NAMs). NAMs can be described as “alternative or complementary methods to or an enhancement of traditional animal testing to predict hazardous properties of chemicals” [110]. NAMs can be split into three general types of method: in silico, in chemico and in vitro.

In silico methods rely on computational models, which can be based on structure activity relationships where a high-speed algorithm searches public databases to identify compounds with a structure similar to that of the test compound. The toxicity of the known compound can then be used to estimate that of the untested one. This is sometimes referred to as ‘read across’ [111]. Another in silico approach is the use of in-vitro-to-in-vivo extrapolation (IVIVE), which is performed by taking in vitro data and applying factors to account for absorption, distribution, metabolism and excretion (ADME). To generate ADME factors, a physiologically based toxicokinetic (PBTK) modelling approach can be applied, which is a mathematical representation of biological processes in the body [112]. Commercial models are available [113], and a guidance document for the development of PBTK models has been published by the OECD [114].

Traditional in vitro methods are not adequate testing systems because 2D cells in wells/flasks receive different physiological signals compared to those in the human body. The development of organoids—organ-specific cell cultures that form 3D tissues representing the organ’s function, structure and complexity—has made in vitro systems more physiologically relevant [115]. Organoids from small intestine, colon, stomach, oesophagus, tongue, liver, lung, pancreas, heart, ear and skin have been described [115]. Assays to evaluate effects of chemicals on the cardiovascular and central nervous systems are also in use as screening assays [116]. However, although an improvement on 2D in vitro cell systems, organoids are grown in Petri dishes and have no blood flow or mechanical stimulation, therefore limiting their relevance to human toxicity. Further development led to organ-on-a-chip, which combines microfluid systems and 3D tissue constructs with cultured human cells to replicate an organ [117]. This model has flowing liquid to supply oxygen and nutrients to the cells in a similar way to that of the human body, resulting in a much more realistic model. The significance of this breakthrough was recognized by the World Economic Forum, which, in 2016, selected organ-on-a-chip as one of the top ten emerging technologies [118].

Initially, one organ was used per chip and models for lung, lymph node, bone marrow, liver, kidney, heart, blood brain barrier, immune system, skin and eye were developed [119].

Although epithelial, Caco-2 cells have been used extensively to measure toxin permeability in traditional in vitro methods, the combination of Caco-2 cells and HT29-MX cells (human mucin-producing goblet cells) yielded a gut-on-a-chip model. This was tested on verapamil, which showed that the permeability determined by the gut-on-a-chip model was consistent with published in vitro values [120]. Combining more than one target organ on a chip has made the technology more powerful, and there have been a huge number of different organ combinations developed [121].

As an extension to this work, research has been conducted to combine greater numbers of organs to create a body-on-a-chip. These systems are used by the pharmaceutical industry in preclinical drug trials as a screen to eliminate drugs associated with toxicity. This means that the number of candidates going forward to animal testing is reduced [122]. However, at this stage, technology cannot fully replace animals, as explained by Warren Casey, the director of the US National Toxicology Program’s Interagency Centre for the Evaluation of Alternative Toxicological Methods, who said in 2019 that “physiology is really, really complex and we still don’t have a handle on it–nor anything that legitimately mimics it aside from animal models” [123]. Issues to be resolved for organ-on-a-chip include the design of a blood surrogate suitable for the co-culture of different cells (organs), improvement in construction materials to ensure that there is no interaction between the device surface and the test compound, the design of an appropriate fluid to create a realistic microenvironment and the scaling of organs to ensure that the ratio of organs in humans is reproduced on the chip [122].

Another barrier to the uptake of organ-on-a-chip technology is its relevance in the regulatory process. Before any test can be used for regulatory purposes, it must be validated and included in official OECD guidelines [124]. A couple of methods, with very simple endpoints, such as “Defined Approaches for Skin Sensitization (OECD TG 497)”, have been accepted, but the acceptance process took 10 years to complete [124]. Regulators must ensure that methods can stand up to any legal challenge [22], and the FDA and Center for Drug Evaluation and Research state “acceptance of any new alternative method will require persuasive evidence that the method improves or otherwise adds value to the current testing strategy and is fit for its intended purpose, so that regulators and the scientific community are confident in the suitability of the new method to inform the safety of human pharmaceuticals and protect human health” [116]. Furthermore, current regulations have been written with animal data in mind, and it is unclear how the complex data generated by new technologies fit into this framework [110].

## 4. Discussion

PSTs in shellfish present a genuine threat to human health, and as such, health-based guidance limits and regulatory limits are required. Shellfish must then be analyzed for the presence of PSTs prior to human consumption using assays that are sufficiently sensitive and able to give an accurate determination of sample toxicity. Ideally, these approaches would be reliable, cheap and fast.

The mouse bioassay, developed in the 1930s, was ingenious in that it assessed the safety of shellfish prior to the cause of toxicity being known. This method safeguarded human health, but as our understanding of PSTs has increased, the weaknesses of this analysis method became apparent. Furthermore, the test was justifiably recognized as being unethical. In some parts of the world, such as Australia, New Zealand and Europe, the MBA has been replaced by analytical methods, but surprisingly, it is still used extensively in other regions, including the United States, Japan and South America. However, although the MBA is used for the testing of shellfish for regulatory purposes, the majority of these areas employ other detection methods for screening. Given the weaknesses of the MBA as well as the availability of other, fully validated analysis methods, it would seem beneficial if approved regulatory methods could be updated.

Over the past 50 years, there has been a massive effort directed toward the development of methods for PST monitoring, which has resulted in a range of diverse techniques being described. Each method has strengths and weaknesses, and it is imperative that the appropriate one is chosen for the specific task. In vitro approaches such as cell based, receptor-based or immunological assays have advantages. For example, they allow high sample throughput without the requirement for expensive equipment, which makes them ideal for sample screening. Commercially available kit assays have been used successfully in the field to allow shellfish to be analyzed prior to harvest. However, while these detection methods can be used successfully for the analysis of a single analyte, the PST class of toxins is very complex, with multiple structurally related analogues existing in nature. Accurate analysis of PSTs using these methods would require their mode of action (e.g., cell death, receptor binding or structure recognition of analogues) to correlate with the toxicity of each analogue. Not surprisingly, it has been shown that this is not the case, which means that while they are of great utility for screening, these assays cannot accurately determine the toxicity of samples. Furthermore, since binding and cross-reactivity do not relate to compound toxicity, it is vital that these data are not used to determine TEFs. In contrast, analytical analysis of shellfish samples allows for the separation of PST analogues such that they can be individually quantified. However, this approach requires expensive equipment, is relatively slow and is reliant on TEFs determined using animal toxicity data. For accuracy, these toxicity data should be determined orally to replicate human exposure, and rather than gavage, voluntary oral consumption should be used. Recent studies have also shown that the experimental protocol, such as the feeding regime, can affect the toxicity of PSTs, so a consistent and defined protocol must be utilized [27].

The 3R rationale to animal testing has been internationally adopted, which has greatly reduced the research capability invested in animal models, with a corresponding increase in the effort to develop alternative methods. Fantastic progress has been made in this area, with the organ-on-a-chip technology representing a major breakthrough. However, despite having great value in the screening and testing of chemicals with relatively simple endpoints, the barriers to body-on-a-chip have not yet been overcome. Therefore, despite early optimism, it is now becoming increasingly accepted that it is impossible to replace animal models that involve complex endpoints. Ofelia Bercaru, the director of prioritization and integration at the European Chemicals Agency stated “The regulatory system regulates chemicals on the basis of adverse effects observed at a particular dose. These effects may be in the structure, function, growth, development or lifespan of an organism. Non-animal methods don’t offer the same observable effects as animals do, making them poor substitutes in complex studies” [124]. One such use of animal models, which cannot be currently replaced, is that used to determine the toxicity of shellfish toxins to allow for the setting of appropriate regulatory limits and for calculation of TEFs. The accuracy of TEFs underpins the analysis of shellfish toxins by analytical methods, which is the sole method capable of a true assessment of shellfish toxicity as required to protect human health.

Using animals to answer only research questions and for the determination of TEFs adheres to the principles of the 3Rs. In New Zealand, animal numbers have been reduced from 80,000/year, when the MBA was used in the screening of shellfish samples, to <400/year being used for research purposes (reduction). These studies also follow OECD guidelines modified to minimize animal numbers and to reduce animal suffering [46], and they avoid gavage dosing, as recommended in the European Union Registration, Evaluation and Authorisation of Chemicals (REACH) legislation [46] (refinement). A partial replacement of animal methods can also be made by the use of in vitro methods for screening (replacement).

## 5. Conclusions

A huge number of methods for the detection of PSTs are currently in use throughout the world. In vitro approaches are best utilized for screening purposes but should not be used in the generation of TEF values. In contrast, the use of animals for routine screening is unnecessary and should be avoided, but they are crucial for determining oral toxicity data for TEF calculations. Only analytical methods, with the application of appropriate TEF values, are capable of yielding accurate PST concentrations for use in regulatory monitoring.

## Figures and Tables

**Table 1:** Toxicity equivalence factors (TEFs) determined for PSTs on different sodium channel subunits [74]. TEFs determined from mouse toxicity data (in vivo tox) by feeding are also displayed. The sodium current TEFs in comparison to those of the mouse toxicity (%) are displayed in red.

PSP Analogue	In Vivo Tox Feeding	Na_v_1.1		Na_v_1.2		Na_v_1.3		Na_v_1.4		Na_v_1.5		Na_v_1.6		Na_v_1.7	
STX	**1**	1		1		1		1		1		1		1	
dcSTX	**0.37**	0.07	19	0.25	68	0.08	22	0.16	43	0.96	259	0.96	259	4.6	1243
NeoSTX	**2.54**	6.4	252	2.6	102	2.9	114	2.6	102	38.6	1520	1.2	47	5	197
dcNeoSTX	**0.2**	0.001	<1	0.1	50	0.024	12	0.001	<1	0.73	365	0.25	125	0.33	165
GTX1&4	**0.94**	0.96	102	0.54	57	1.6	170	0.57	61	14.4	1530	1.4	149	9.29	988
GTX5	**0.06**	0.015	3	0.014	23	0.08	133	0.18	300	10.7	1780	0.11	183	4.08	680
GTX2&3	**0.57**	0.2	35	0.39	68	0.64	112	0.32	56	3.87	679	0.15	26	0.27	47
dcGTX2&3	**0.11**	0.04	36	0.05	45	0.22	200	0.01	9	3.3	300	0.02	182	3.1	282
C1&2	**0.04**	0.008	20	0.013	33	0.25	625	0	0	2.6	650	0.09	225	0.1	250

**Table 2:** Cross-reactivities of PSTs in relation to STX determined by ELISA or LFA. TEFs determined from mouse toxicity (in vivo tox) data by feeding are also displayed. The cross-reactivities in comparison to the mouse toxicity TEF (%) are displayed in red.

PST Analogue	In Vivo Tox Feeding	Abraxis ELISA		Europroxima ELISA		R-Biopharm ELISA		Beacon ELISA		Bioo ELISA		Scotia LFA		Neogen LFA	
STX	**1**	1		1		1		1		1		1		1	
dcSTX	**0.37**	0.29	78	0.19	51	0.2	54	0.18	49	1	100	0.71	192	0.56	151
NeoSTX	**2.54**	0.013	<1	0.014	<1	0.12	<1	0.08	3	0.2	8	0.26	10	1.29	51
dcNeoSTX	**0.2**	0.006	3	0.005	3			0.007	4	0.04	20			0.28	140
GTX1&4	**0.94**	0.002	<1	0.001	<1			0.001	<1	0.02	2	0.03	3	0.06	6
GTX5	**0.06**	0.23	383	0.26	433			0.26	433	0.61	1017	0.57	950	0.23	383
GTX2&3	**0.57**	0.3	53	0.056	10	0.7	123	0.12	<1	0.1	18	0.8	140	0.23	40
dcGTX2&3	**0.11**	0.014	13	0.002	2			0.004	4	0.43	391	0.16	145	0.08	73
C1&2	**0.04**	0.002	5	0.002	5	0.0	0	0.014	35			0.1	250	0.03	75

## Data Availability

No new data were created or analyzed in this study.
